# Polygenic height prediction for the Han Chinese in Taiwan

**DOI:** 10.1038/s41525-025-00468-6

**Published:** 2025-02-05

**Authors:** Chih-Hao Chang, Che-Yu Chou, Timothy G. Raben, Shih-Ann Chen, Yuh-Jyh Jong, Jeng-Yih Wu, Shun-Fa Yang, Hsiang-Cheng Chen, Yen-Lin Chen, Ming Chen, Gwo-Chin Ma, Chih-Yang Huang, Tso-Fu Wang, Sing-Lian Lee, Chen-Fang Hung, See-Tong Pang, Erik Widen, Yao-Ming Chang, Erh-Chan Yeh, Chun-Yu Wei, Chien-Hsiun Chen, Stephen D. H. Hsu, Pui-Yan Kwok

**Affiliations:** 1 Institute of Biomedical Sciences, Academia Sinica, Taipei, Taiwan; 2 Department of Physics and Astronomy, Michigan State University, East Lansing, Michigan USA; 3 Department of Post-Baccalaureate Medicine, College of Medicine, National Chung Hsing University, Taichung, Taiwan; 4 Cardiovascular Center, Taichung Veterans General Hospital, Taichung, Taiwan; 5 Heart Rhythm Center, Division of Cardiology, Department of Medicine, Taipei Veterans General Hospital, Taipei, Taiwan; 6 Chair Professor of Graduate Institute of Clinical Medicine, College of Medicine, Kaohsiung Medical University (KMU), Kaohsiung, Taiwan; 7 Visiting Staff, Departments of Pediatrics and Laboratory Medicine, Kaohsiung Medical University Hospital, Kaohsiung, Taiwan; 8 Former President of Kaohsiung Medical University, Kaohsiung, Taiwan; 9 President, Taiwan SMA Families, Kaohsiung, Taiwan; 10 Health Management Center, Department of Gastroenterology, Kaohsiung Medical University Hospital, Kaohsiung Medical University, Kaohsiung, Taiwan; 11 Institute of Medicine, Chung Shan Medical University, Taichung, Taiwan; 12 Department of Medical Research, Chung Shan Medical University Hospital, Taichung, Taiwan; 13 Division of Rheumatology/Immunology and Allergy, Department of Internal Medicine, Tri‑Service General Hospital, National Defense Medical Center, Taipei, Taiwan; 14 Center for Precision Medicine and Genomics, 2. Department of Pathology, Tri-Service General Hospital, National Defense Medical Center, Taipei, Taiwan; 15 Department of Genomic Medicine, Changhua Christian Hospital, Changhua, Taiwan; 16 Cardiovascular and Mitochondria Related Disease Research Center, Hualien Tzu Chi Hospital, Buddhist Tzu Chi Medical Foundation, Hualien, Taiwan; 17 Department of Medical Research, China Medical University Hospital, China Medical University, Taichung, Taiwan; 18 Center of General Education, Buddhist Tzu Chi Medical Foundation, Tzu Chi University of Science and Technology, Hualien, Taiwan; 19 Graduate Institute of Basic Medical Science, China Medical University, Taichung, Taiwan; 20 Department of Hematology and Oncology, Hualien Tzu Chi Hospital, Buddhist Tzu Chi Medical Foundation, Hualien, Taiwan; 21 Department of Hematology and Oncology, School of Medicine, Tzu Chi University, Hualien, Taiwan; 22 Division of Endocrinology, Department of Internal Medicine, Koo Foundation Sun Yat-Sen Cancer Center, Taipei, Taiwan; 23 Department of Research, Koo Foundation Sun Yat-Sen Cancer Center, Taipei, Taiwan; 24 Chang Gung Memorial Hospital at Linkou, Taoyuan City, Taiwan; 25 Chang Gung University, Taoyuan City, Taiwan; 26 Genomic Prediction, North Brunswick, New Jersey, USA; 27 Present Address: Department of Medicine, University of California San Francisco, San Francisco, CA USA; 28 Present Address: Cardiovascular Research Institute, Institute for Human Genetics, and Department of Dermatology, University of California San Francisco, San Francisco, CA USA

**Keywords:** Genetics, Heritable quantitative trait

## Abstract

Human height prediction based on genetic factors alone shows positive correlation, but predictors developed for one population perform less well when applied to population of different ancestries. In this study, we evaluated the utility of incorporating non-genetic factors in height predictors for the Han Chinese population in Taiwan. We analyzed data from 78,719 Taiwan Biobank (TWB) participants and 40,641 Taiwan Precision Medicine Initiative (TPMI) participants using genome-wide association study and multivariable linear regression least absolute shrinkage and selection operator (LASSO) methods to incorporate genetic and non-genetic factors for height prediction. Our findings establish that combining birth year (as a surrogate for nutritional status), age at measurement (to account for age-associated effects on height), and genetic profile data improves the accuracy of height prediction. This method enhances the correlation between predicted and actual height and significantly reduces the discrepancies between predicted and actual height in both males and females.

## Introduction

Human height is a widely studied polygenic trait because it can be measured accurately and is readily available from large cohorts^1–4^. Besides sex differences and genetic factors, however, adult height is also influenced by nutrition, age, and environmental factors. For example, from 1985 to 2019, the average height of males and females in Taiwan increased from 169.2 cm to 173.5 cm and from 158.3 cm to 160.7 cm, respectively; those for males and females in the United Kingdom increased from 176.4 cm to 178.2 cm and from 162.7 cm to 163.9 cm, respectively^5–10^. In addition, height is associated with several human diseases, including cancer^11,12^, coronary heart disease^13^, stroke^14^, and macular degeneration^15^. It is speculated that genetic loci associated with height may be pleiotropic and influence one’s susceptibility to diseases. Genome-wide association studies (GWAS) and machine learning techniques have been used to identify genetic variants associated with height^16–22^.

Polygenic prediction of height has been examined extensively in European populations^19,23,24^ and briefly in admixed populations^25,26^, but compared with studies in European populations, fewer studies have examined polygenic height prediction in non-European populations^27^. Here, we report our findings of height prediction based on genetic and other factors in two large Han Chinese cohorts as part of the Taiwan Biobank (78,719 individuals) and the Taiwan Precision Medicine Initiative (40,641 individuals).

## Methods

### Sample characteristics

The Taiwan Biobank (https://www.twbiobank.org.tw/; TWB) is a community-based prospective community cohort study of the Taiwanese population. Those between 30 to 70 years old and without cancer can join the project, but there is no age limitation for tracking cases. A standard questionnaire is used at 44 recruitment centers in all counties and cities across Taiwan to collect participants’ demographics, socioeconomic status, environmental exposures, lifestyle, dietary habits, family history and self-reported disease status. Anthropometric measurements and blood / urine samples are collected at the time of enrollment, and genetic profiling is performed on custom genome-wide single nucleotide polymorphism (SNP) arrays, TWBv1 with 653,291 SNPs and TWBv2 (also named TPM1) with 752,921 SNPs^28^.

The Taiwan Precision Medicine Initiative (TPMI; https://tpmi.ibms.sinica.edu.tw/www/) is a cohort study of the Taiwanese population in partnership with 33 hospitals across Taiwan. Participants consent to providing their electronic medical records (EMR) and residual blood samples for genetic profiling. The EMR includes outpatient and admission/discharge notes, surgical records, together with imaging, pathological, and blood test reports. Genetic profiling was performed on two custom genome-wide SNP arrays. The first array, TPM1, also known as TWBv2, has 752,921 SNPs and was used for early participants (before 2022). The second array, TPM2, has 755,191 SNPs and was used for subsequent participants^29^.

This study includes 81,061 TWB participants genotyped on the TPM1/TWBv2 arrays as a training and testing set, and 68,610 TPMI participants genotyped on the TPM1/TWBv2 arrays as a validating set and for subsequent analysis. The latest height measurements are used in the analyses for those TWB and TPMI participants with follow-up data.

Written informed consent was obtained from all participants, with ethics approval granted by the Academia Sinica Institutional Review Board (AS-IRB01-23066). This study was conducted in accordance with the Declaration of Helsinki and relevant ethical regulations. Summary statistics are available from the corresponding author upon reasonable request, and individual data and biomaterials can be accessed through the Taiwan Biobank following established procedures.

### Quality control

We conducted standard quality control (QC) for the two datasets^30–32^ (Supplementary Figure 1). Individuals with gender error, genotyping miss call rate > 0.1, birthdate outside 1946–1986 range, and 3rd degree or closer kinship relationship with other participants were excluded, resulting in 78,719 QC-passed samples from 81,061 TWB participants in the training and testing sets, and 40,641 QC-passed samples passed from 68,610 TPMI participants in the validating set. Next, we removed SNPs with call rates of <0.9, minor allele frequency (MAF) < 0.01, and deviation from Hardy-Weinberg equilibrium (HWE < 10-8), resulting in 543,064 and 543,701 high quality SNPs in the training/testing and validating sets, respectively. Finally, 542,988 SNPs in common between the training/testing and validating sets were selected for subsequent analyses.

### Statistical analysis

Males and females are analyzed separately due to known height differences between them. Height predictors are developed using the following process (Supplementary Figure 2): (I) We employed the “10-Fold Cross-Validation” method^33–36^ to randomly divide all TWB samples into 10 subgroups, labeled G1, G2, …, G9, and G10. (II) When the G1 subgroup was used as the testing set then the other 9 subgroups, G2-G10, were used as the training set. Similarly, G2 was used as the testing set and the other 9 subgroups, G1, G3-G10, were used as the training sets and so on. In this step, 10 analysis groups of training and testing sets were obtained. (III) The genome-wide association study (GWAS) was conducted by regressing height (dependent variable) on the single nucleotide polymorphism (SNP) (independent variable), one SNP at a time, on the training set in each analysis group. Manhattan plot results for males and females were presented in Supplementary Figure 3. (IV) Next, we filtered out SNPs with *P*-values greater than 0.05 in the 10 analysis group training sets and further selected the intersecting SNPs that were present in all 10 groups for subsequent analysis. (V) To select a subset of informative SNPs that illustrate the relationship between the genome and height, the maximum R-square stepping algorithm least absolute shrinkage and selection operator (LASSO)^37^ method was used (via a least angle regression) in the 10 analysis group training sets. Only SNP information was included to select the most appropriate SNP combination. (VI) We then picked out the intersecting SNPs that were selected by the step (V) LASSO method in all the 10 analysis groups training set. There were 5,878 SNPs in the male groups and 20,311 SNPs in the female groups in the final combination of SNPs (Supplementary Table 1). (VII) Multiple linear regression was used to calculate the weights of four different combinations in the 10 analysis groups training set: SNPs (polygenic score, PGS) only, SNPs (PGS) + birth year in Anno Domini (AD), SNP (PGS) + age at measurement, and SNP (PGS) + birth year in AD + age at measurement. The weight, beta value, of each SNP, birth year in AD, and age at interview were used for subsequent height prediction. (VIII) We used the weights from step (VII) to calculate the average of the predicted heights over 10 runs for the TWB training and testing sets and, TPMI validating sets, respectively. The descriptive statistics of height and age, mean, standard deviation (SD), median, and range are presented in TWB training and testing and TPMI validating sets. All data analyses were performed using PLINK^30^, KING^38^, SAS 9.4 (SAS institute, Cary, NC, USA), and R 4.2.2 (R Foundation for Statistical Computing, Vienna, Austria).

## Results

### Clinical Characteristics of Taiwan Biobank (TWB) and Taiwan Precision Medicine Initiative (TPMI)

**Fig. 1:**
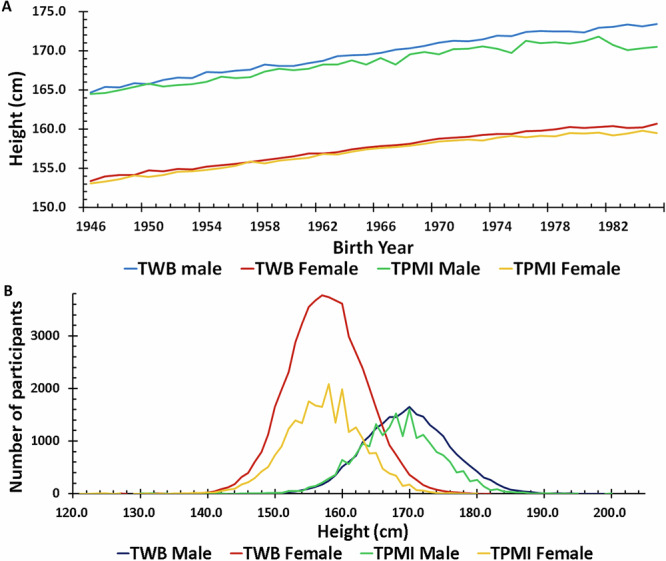
Taiwan Biobank and Taiwan Precision Medicine Initiative (TPMI) females’ and males’ height distribution. **A** The average height in the year of birth, the trendline equation: TWB Male = 0.2258*(year of birth in AD)-274.22; TPMI Male = 0.1789*(year of birth in AD)-183.20; TWB Female = 0.1890*(year of birth in AD)-213.90; TPMI Female = 0.1766*(year of birth in AD)-190.01, **B** count of the different heights.

After performing quality control measures, a total of 119,360 individuals are included in this study (TWB: 54,064 females and 24,655 males; TPMI: 22,508 females and 18,133 males). There is no significant difference between the distribution of height in the TWB and TPMI datasets (Table 1 and Fig. 1B). Tabulation of the average height based on birth year (between 1946 and 1986) clearly shows an increase in average height in participants born in later years (Fig. 1A). The trendline slopes of the height are: 0.2258 in TWB males, 0.1890 in TWB females, 0.1789 in TPMI males and 0.1766 in TPMI females, respectively. Birth year is therefore included in the model to account for the effect of nutritional and other improvement in Taiwan between 1946 and 1986 on height. As height also varies with age, age at measurement is included in the model to account for this.

**Table 1:** Clinical characteristic of Taiwan Biobank (TWB) and Taiwan Precision Medicine Initiative (TPMI) participants

	TWB		TPMI	
	Female	Male	Female	Male
**Number**	54064	24655	22508	18133
**Age at measurement (years)**				
** Mean** **±** **SD**	51.68 ± 10.40	51.96 ± 11.16	53.09 ± 12.15	55.90 ± 11.64
** Median**	52.91	53.02	54.03	57.94
** Range**	30.00–73.79	30.00–73.57	30.00–74.99	30.01–74.97
*** P*-value**^**a**^	-	-	<0.0001	<0.0001
**Height (cm)**				
** Mean** **±** **SD**	157.43 ± 5.66	169.47 ± 6.29	157.17 ± 5.83	168.05 ± 6.80
** Median**	157.50	169.50	157.00	168.00
** Range**	118.50–181.50	112.00–200.00	124.00–200.00	127.50–202.50
*** P*-value**^**a**^	-	-	0.6336	0.1505

^*a*^*P*-value for *t*-test compared to TWB dataset.

### Actual height vs. predicted height in TWB training and testing sets

**Fig. 2:**
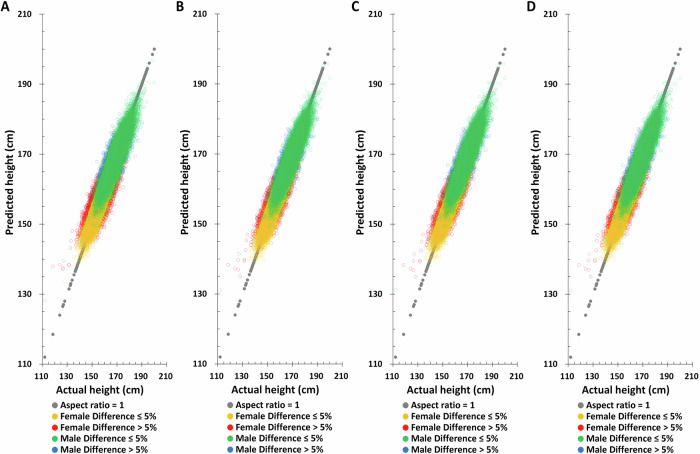
The distribution of actual height and predicted height based on the combination of different factors combination in the Taiwan Biobank training set. **A** Polygenic score (PGS) only, **B** PGS + birth year in AD, **C** PGS + age at measurement, **D** PGS + birth year in AD + age at measurement.

Birth year, age at measurement, SNPs obtained by the univariable linear regression, and their weights obtained by the multivariable linear regression LASSO selection method are used to predict height in the TWB training set. Using only birth year, only age at measurement, or birth year plus age at measurement to predict height does not yield good predictions in the TWB training set (Supplementary Figure 4 and Supplementary Table 2) but adding height-related SNPs increases height prediction accuracy (Fig. 2A–C and Table 2). Combining birth year, age at measurement, and height-related SNPs simultaneously improves the accuracy of height prediction and decreases difference between actual and predicted height (Fig. 2D and Table 2).

**Table 2:** The actual and predicted height distribution in TWB participants (*N* = 78,719)

Male						
Model	*N*	Mean ± SD	Median (range)	Difference > 5% N (%)^a^	PCC	PCC’s SD
**Actual**						
	24655	169.47 ± 6.29	169.5 (112.00–200.00)			
**Training set**						
**PGS**	221895	169.47 ± 5.40	169.38 (128.19–196.72)	179 (0.73%)	0.8675	0.0011
**PGS** **+** **birth year in AD**	221895	169.47 ± 5.50	169.41 (131.08–195.88)	133 (0.54%)	0.8825	0.0010
**PGS** **+** **age at measurement**	221895	169.47 ± 5.50	169.41 (131.18–195.84)	134 (0.54%)	0.8825	0.0010
**PGS** **+** **birth year in AD** **+** **age at measurement**	221895	169.47 ± 5.50	169.40 (131.13–195.86)	132 (0.54%)	0.8826	0.0010
**Testing set**						
**PGS**	24655	169.48 ± 5.61	169.40 (138.82–200.43)	1184 (4.80%)	0.7481	0.0042
**PGS** **+** **birth year in AD**	24655	169.47 ± 5.69	169.41 (140.59–199.00)	924 (3.75%)	0.7758	0.0040
**PGS** **+** **age at measurement**	24655	169.47 ± 5.69	169.41 (140.70–199.16)	922 (3.74%)	0.7757	0.0040
**PGS** **+** **birth year in AD** **+** **age at measurement**	24655	169.47 ± 5.69	169.40 (140.62–199.04)	920 (3.73%)	0.7759	0.0040

Female						
Model	*N*	Mean ± SD	Median (range)	Difference > 5% N (%)^a^	PCC	PCC’s SD
**Actual**						
	54064	157.43 ± 5.66	157.50 (118.50–181.50)			
**Training set**						
**PGS**	486576	157.44 ± 4.75	157.38 (133.77–179.61)	497 (0.92%)	0.8509	0.0008
**PGS** **+** **birth year in AD**	486576	157.44 ± 4.88	157.39 (135.06–180.68)	301 (0.56%)	0.8717	0.0007
**PGS** **+** **age at measurement**	486576	157.44 ± 4.88	157.38 (134.94–180.59)	295 (0.55%)	0.8718	0.0007
**PGS** **+** **birth year in AD** **+** **age at measurement**	486576	157.43 ± 4.88	157.38 (135.04–180.66)	294 (0.54%)	0.8719	0.0007
**Testing set**						
**PGS**	54064	157.44 ± 5.47	157.41 (108.54–244.86)	7144 (13.21%)	0.5427	0.0036
**PGS** **+** **birth year in AD**	54064	157.44 ± 5.51	157.41 (106.93–242.99)	5784 (10.70%)	0.6032	0.0034
**PGS** **+** **age at measurement**	54064	157.44 ± 5.51	157.42 (104.26–243.81)	5745 (10.63%)	0.6026	0.0034
**PGS** **+** **birth year in AD** **+** **age at measurement**	54064	157.43 ± 5.48	157.41 (111.32–209.12)	5722 (10.58%)	0.6084	0.0034

*PGS* polygenic score, *SD* standard deviation, *AD* Anno Domini, *PCC* Pearson correlation coefficient, SD equation for PCC = sqrt [(1 - PCC²) / (N - 2)].

^a^ Number of samples with a value >0.05 from take the absolute value after subtracting the actual height from the predicted height and then divided by the actual height.

**Fig. 3:**
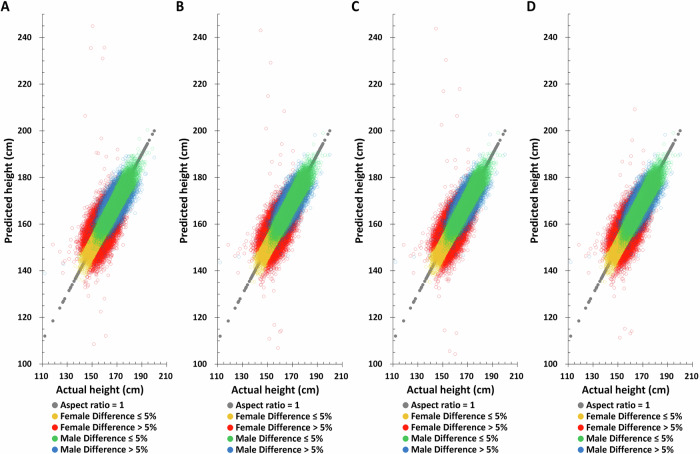
The distribution of actual height and predicted height based on the combination of different factors combination in the Taiwan Biobank testing set. **A** PGS only, **B** PGS + birth year in AD, **C** PGS + age at measurement, **D** PGS + birth year in AD + age at measurement.

Applying the same method to predict height in the TWB testing set, the Pearson correlation coefficient between actual and predicted height based on birth year, age at measurement, and height-related SNPs are found to be 0.7759 and 0.6084 for males and females, respectively (Table 2), substantially higher than all other combinations. Moreover, the difference between the height predicted by this combination and the actual height is also the smallest. The distribution of actual and predicted height in the TWB testing set also yields the same results, suggesting that combining birth year, age at measurement, and height-related SNPs will give the best height predictions (Fig. 3 and Supplementary Fig. 5).

### Actual height vs. principal component analysis (PCA) adjustment predicted height in TWB training and testing sets

**Fig. 4:**
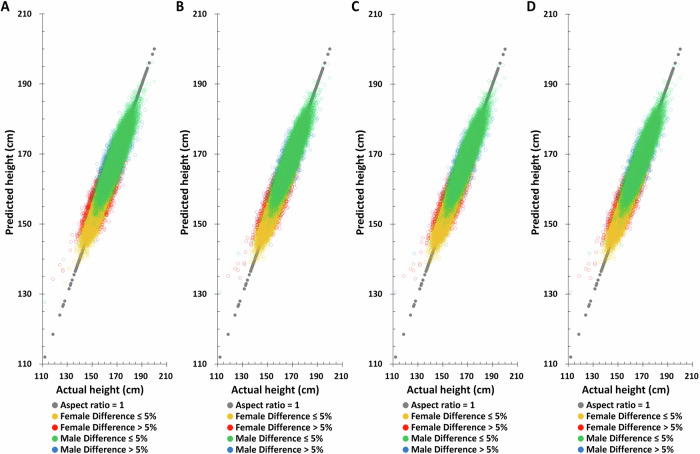
The distribution of actual height and principal component analysis (PCA) adjustment predicted height based on the combination of different factors combination in the Taiwan Biobank training set. **A** PGS only, **B** PGS + birth year in AD, **C** PGS + age at measurement, **D** PGS + birth year in AD + age at measurement.

**Fig. 5:**
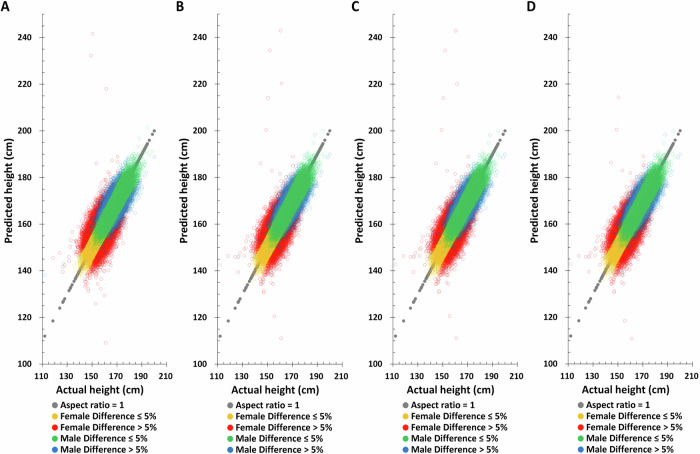
The distribution of actual height and principal component analysis (PCA) adjustment predicted height based on the combination of different factors combination in the Taiwan Biobank testing set. **A** PGS only, **B** PGS + Birth year in AD, **C** PGS + Age at measurement, **D** PGS + Birth year in AD + Age at measurement.

In most genomic related studies, the principal component analysis (PCA) adjustment is applied to correct for population stratification^19,23,25,27,28^. The TWB female and male PCA eigenvalues were tabulated in Supplementary Table 1. Since the PCA eigenvalue after the 20th in females is <1, PCA1-PCA20 were selected for subsequent analysis. Although males are <1 from the 15th onwards, based on the consistency of analysis, the same PCA1-PCA20 as females were selected for subsequent analysis (Supplementary Table 3). The outcomes are the same as those without 20 PCA factors (Figs. 4, 5, and Table 3). Though the accuracy was more precise, there were no statistically significant differences between the TWB testing set without and with PCA adjustment; the Pearson correlation coefficient in males was 0.7759 and 0.7816, respectively. And in the female group, it was 0.6084 and 0.6262 (Tables 2 and 3). The overall impact of PCA is minimal. In the subsequent validation analysis, we opted for a model that excluded PCA to mitigate the potential variations stemming from differing PCA coefficients across various databases.

**Table 3:** The actual and principal component analysis (PCA) adjustment predicted height distribution in TWB participants (*N* = 78,719)

Male						
Model	*N*	Mean ± SD	Median (range)	Difference > 5% N (%)^a^	PCC	PCC’s SD
**Actual**						
	24655	169.47 ± 6.29	169.5 (112.00–200.00)			
**Training set**						
**PGS** + **PC1 - PC20**	221895	169.47 ± 5.43	169.38 (127.70–196.31)	151 (0.61%)	0.8726	0.0010
**PGS** **+** **birth year in AD** + **PC1 - PC20**	221895	169.47 ± 5.52	169.38 (130.53–195.57)	119 (0.48%)	0.8856	0.0010
**PGS** **+** **age at measurement** + **PC1 - PC20**	221895	169.47 ± 5.52	169.39 (130.62–195.53)	118 (0.48%)	0.8857	0.0010
**PGS** **+** **birth year in AD** **+** **age at measurement** + **PC1 - PC20**	221895	169.47 ± 5.52	169.39 (130.58–195.55)	117 (0.47%)	0.8858	0.0010
**Testing set**						
**PGS** + **PC1 - PC20**	24655	169.48 ± 5.64	169.39 (138.11–201.41)	1132 (4.59%)	0.7573	0.0042
**PGS** **+** **birth year in AD** + **PC1 - PC20**	24655	169.48 ± 5.71	169.42 (139.55–199.92)	864 (3.50%)	0.7815	0.0040
**PGS** **+** **age at measurement** + **PC1 - PC20**	24655	169.47 ± 5.71	169.41 (139.66–200.07)	867 (3.52%)	0.7814	0.0040
**PGS** **+** **birth year in AD** **+** **age at measurement** + **PC1 - PC20**	24655	169.48 ± 5.71	169.41 (139.59–199.96)	862 (3.50%)	0.7816	0.0040

Female						
Model	*N*	Mean ± SD	Median (range)	Difference > 5% N (%)^a^	PCC	PCC’s SD
**Actual**						
	54064	157.43 ± 5.66	157.50 (118.50–181.50)			
**Training set**						
**PGS** + **PC1 - PC20**	486576	157.44 ± 4.81	157.39 (133.26–178.97)	387 (0.72%)	0.8596	0.0007
**PGS** **+** **birth year in AD** + **PC1 - PC20**	486576	157.44 ± 4.92	157.39 (134.56–180.08)	244 (0.45%)	0.8778	0.0007
**PGS** **+** **age at measurement** + **PC1 - PC20**	486576	157.44 ± 4.92	157.38 (134.45–180.00)	242 (0.45%)	0.8779	0.0007
**PGS** **+** **birth year in AD + age at measurement** + **PC1 - PC20**	486576	157.44 ± 4.92	157.38 (134.54–180.07)	239 (0.44%)	0.8780	0.0007
**Testing set**						
**PGS** + **PC1 - PC20**	54064	157.43 ± 5.47	157.40 (109.07–241.61)	6606 (12.22%)	0.5711	0.0035
**PGS** **+** **birth year in AD** + **PC1 - PC20**	54064	157.43 ± 5.51	157.42 (111.06–242.96)	5345 (9.89%)	0.6229	0.0034
**PGS** **+** **age at measurement** + **PC1 - PC20**	54064	157.44 ± 5.52	157.42 (109.89–237.99)	5360 (9.91%)	0.6211	0.0034
**PGS + birth year in AD** **+** **age at measurement** + **PC1 - PC20**	54064	157.43 ± 5.48	157.42 (110.86–214.27)	5311 (9.82%)	0.6262	0.0034

*PGS* polygenic score, *SD* standard deviation, *AD* Anno Domini, *PCC* Pearson correlation coefficient, SD equation for PCC = sqrt [(1 - PCC²) / (N - 2)].

^**a**^ Number of samples with a value greater than 0.05 from take the absolute value after subtracting the actual height from the predicted height and then divided by the actual height.

### Validation of height predictions in the TPMI dataset

**Fig. 6:**
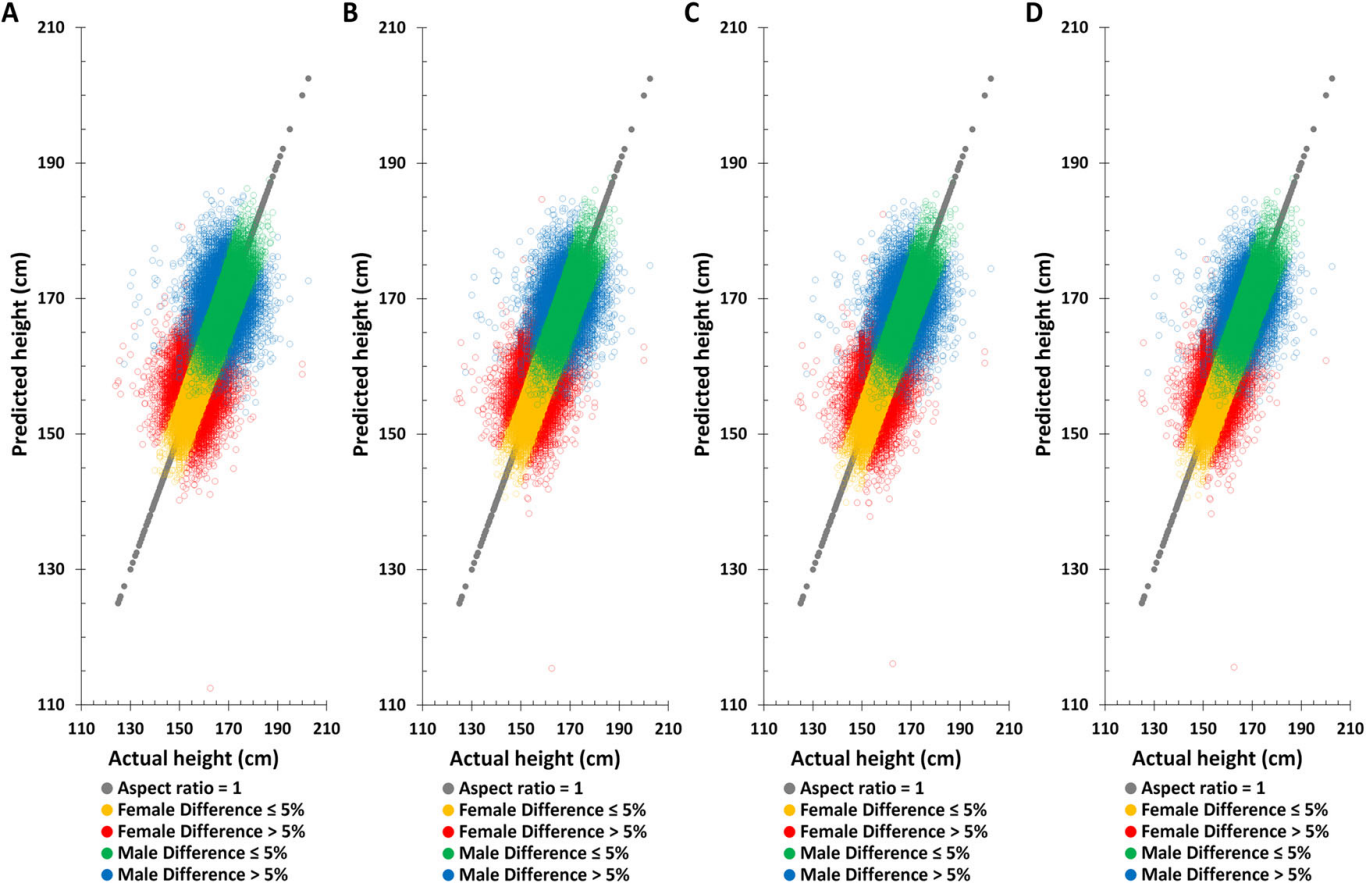
The distribution of actual height and predicted height based on the combination of different factors combination in the TPMI validating set. **A** PGS only, **B** PGS + birth year in AD, **C** PGS + age at measurement, **D** PGS + birth year in AD + age at measurement.

To assess the reliability of the height prediction method, an independent dataset, TPMI, was employed for validation and performance evaluation. Birth year, age at measurement, and height-related SNPs with the same weighting was used for height prediction in the TPMI dataset. The distribution of actual and predicted height based on the combination model (birth year, age at measurement, and height-related SNPs) again shows improvement in the accuracy of height prediction compared to those using only one element or combinations of only two of the elements (Fig. 6). For example, the combination model improves the correlation of predicted to actual height from 0.2225 to 0.3980 for males and 0.2708 to 0.4444 for females, respectively (Table 4). Similarly, the proportion of males and females with >5% difference between their actual and predicted heights decreases significantly, from 19.51% to 11.70% for males and from 19.93% to 13.20% for females, respectively.

**Table 4:** The actual and predicted height distribution in TPMI participants (*N* = 40,641)

Male									
Variable	Model	*N*	Mean ± SD	Median	Minimum	Maximum	Difference > 5% N (%)^a^	PCC	PCC’s SD
**Actual**		18133	168.05 ± 6.80	168.00	127.50	202.50			
**Predicted**	**PGS**	18133	169.52 ± 4.26	169.53	153.18	187.49	3537 (19.51%)	0.2225	0.0072
	**PGS** **+** **birth year in AD**	18133	169.34 ± 4.21	169.32	154.36	187.84	2723 (15.02%)	0.3476	0.0070
	**PGS** **+** **age at measurement**	18133	168.97 ± 4.20	168.94	153.93	187.47	2634 (14.53%)	0.3455	0.0070
	**PGS** **+** **birth year in AD** **+** **age at measurement**	18133	169.18 ± 4.17	169.15	154.20	187.71	2122 (11.70%)	0.3980	0.0068

Female									
Variable	Model	*N*	Mean ± SD	Median	Minimum	Maximum	Difference > 5% N (%)^a^	PCC	PCC’s SD
**Actual**		22508	157.17 ± 5.83	157.00	124.00	200.00			
**Predicted**	**PGS**	22508	157.33 ± 4.25	157.31	112.45	180.53	4485 (19.93%)	0.2708	0.0064
	**PGS** **+** **birth year in AD**	22508	157.58 ± 4.41	157.55	115.44	184.67	3538 (15.72%)	0.4035	0.0061
	**PGS** **+** **age at measurement**	22508	157.11 ± 4.42	157.07	116.09	182.44	3550 (15.77%)	0.3991	0.0061
	**PGS** **+** **birth year in AD** **+** **age at measurement**	22508	157.49 ± 4.38	157.45	115.56	175.78	2972 (13.20%)	0.4444	0.0060

*PGS* polygenic score, *SD* standard deviation, *AD* Anno Domini, *PCC* Pearson correlation coefficient, SD equation for PCC = sqrt [(1 - PCC²) / (*N* - 2)].

^a^ Number of samples with a value >0.05 from take the absolute value after subtracting the actual height from the predicted height and then divided by the actual height.

## Discussion

Geographical location and environmental factors influence Taiwan’s population composition. The majority of Taiwanese ethnic groups are of Han ancestry (>95%), with ~2% being of Aboriginal ancestry (Austronesian)^39,40^. Furthermore, based on PCA results comparing TWB and TPMI samples with the 1000 Genomes Project, the TWB and TPMI samples cluster with East Asian ancestry, confirming that the majority of the samples belong to the Han Chinese ancestry group (Supplementary Fig. 6). The Taiwanese Han Chinese population comprises Min-Nan (also known as Holo), Hakka, and Mainlanders. Although there are genetic, lifestyle, and dietary habit differences among these ethnicities, there is no statistically significant difference in actual and predicted height when comparing the three major ethnic groups (ethnic information comes from TWB phenotypic data) within the Taiwanese Han Chinese population (Supplementary Table 4).

Generally, humans grow taller and consume more nutritious diets when food is abundant^41^. However, as age increases, height tends to gradually decrease due to factors such as spinal disc degeneration, osteoporosis, and muscle loss. Women typically lose around two inches between the ages of 30 and 70, while men lose about an inch by age 70 and two inches by age 80^42–46^. This is consistent with the results shown in this study, where bone density increased with birth year in both males and females but decreased with age at measurement (Supplementary Table 5). Although the inclusion of both birth year in AD and age at measurement in the prediction model raises concerns about collinearity and potential overfitting, our analysis indicates that the variance inflation factors (VIF) for birth year in AD and age at measurement were 9.36 and 8.72, respectively, both below the threshold of 10, indicating no collinearity issues. Therefore, including birth year in AD and age at measurement in the model increases the accuracy of height prediction.

The average age at measurement for the TPMI validating set (female: 53.09 ± 12.15; male: 55.90 ± 11.64) is slightly older than the TWB training and testing sets (female: 51.68 ± 10.40; male: 51.96 ± 11.16) for both sexes. This difference may cause a bias for the height prediction. However, age at measurement is included in the correction in analysis models to avoid the impact of differences. With the additional adjustment, we can also estimate the impact of birth cohort changes on height by using the deviation caused by birth year. The new method for height prediction that combines genetic and age factors as a surrogate for nutritional status in two large datasets (TWB and TPMI), is shown to estimate height accurately for the Han Chinese in Taiwan.

In our analysis, all 10 analysis groups (G1-G10) were used simultaneously as both training and testing sets. This dual role of the data could potentially lead to the testing set showing a somewhat inflated performance due to its inclusion in the training process. This observation may explain the notably high Pearson correlation coefficient of 0.7759 for male and 0.6084 for female observed in the model involving SNPs, birth year, and age at measurement in the TWB male testing set. However, while the result on the testing set may be somewhat inflated due to its dual role in the analysis, the independent TPMI dataset validation results remain robust and are the primary focus of our paper. That said, we acknowledge that there is a notable drop in the correlation coefficient, decreasing from over 0.7 in the testing set to ~0.4 in the validation set. This reduction highlights the challenges of generalizing the model to an independent dataset. The validation results provide a more reliable assessment of the model’s generalization performance, which is consistent with other articles^47,48^ and constitutes a key aspect of our findings.

Furthermore, we observed that females required a larger set of SNPs (20,311 SNPs) compared to males (5878 SNPs) to achieve higher prediction accuracy. One plausible explanation is the influence of hormonal dynamics, particularly estrogen levels, which play a significant role in skeletal growth and development. Hormonal fluctuations, such as those occurring during menopause^49,50^, can impact height-related genetic variants differently in females compared to males. Additionally, age-related processes, including height loss due to aging, may necessitate the inclusion of a broader array of genetic markers in females to account for these physiological changes.

A recent study analyzed the rare and low-frequency coding variants found in >200,000 individuals of six different ethnicities and identified >1000 variants associated with height^51^. The authors observed that these variants were associated with body mass index, bone mineral density, and lung function^51^. In a GWAS study of the Taiwan Biobank (TWB), four novel genes—NABP2, RASA2, RNF41, and SLC39A5—were identified for human height, and it was also discovered that these genes have associated with cardiovascular disease, diabetes, and cancer^52^. In our current study, with the exception of rs295321 in the RASA2 gene, all SNPs from these four height-related genes (NABP2, RASA2, RNF41, and SLC39A5) were incorporated into our height prediction model. Other studies using TWB data have suggested potential associations between height and certain health-related outcomes, though the most significant findings were related to anthropometric traits. While there may be trends indicating that taller individuals could have lower risks for some chronic diseases, such as cardiovascular disease, diabetes, and cancer, these associations are not definitive. Additionally, height has been suggested to be associated with longer life expectancy in some populations, but further research is needed to confirm these findings^53^. In addition, the relationship between height and mate choice and reproduction in Taiwan found that taller men were more likely to have a partner and have more children. They were also more likely to have shorter periods of celibacy and live with their partners for longer periods of their lives^54^. Furthermore, a polygenic risk predisposition score for familial short stature (FSS) in the Han Chinese population, comprising 10 novel SNPs and nine previously reported height-related SNPs, demonstrated high predictive accuracy for FSS risk, with an area under the curve of 0.940 in the testing group^55^. These nine height-related SNPs have also been included in our height prediction model to enhance its predictive capability. The height prediction study by Yengo et al.^16^ identified 12,111 independent SNPs significantly associated with height, based on data from a genome-wide association study of 5.4 million individuals from diverse ancestries^16^. In our analysis, 34.20% ((855 + 1155)/5878) of these SNPs were included in our male height prediction model, and 33.55% ((1155 + 5659)/20311) were included in our female model (supplementary Fig. 7). Although approximately one-third of the SNPs overlap, the heritability (***h²***) of the SNPs we selected for height prediction is 0.4775 ± 0.0069 in males and 0.4267 ± 0.0050 in females. Furthermore, gene ontology (GO) analysis based on the SNPs we selected for height prediction identified the top 30 GO terms with the smallest false discovery rate (FDR) (Supplementary Fig. 8). The top five GO terms in females—developmental process, system development, anatomical structure development, multicellular organism development, and multicellular organismal process—are all related to height (Supplementary Fig. 8A). Similarly, in males, the GO term with the smallest FDR is also related to height (Supplementary Fig. 8B). Therefore, the SNPs we selected are indeed associated with height and can be used for height prediction.

Limitations of this study include the following: First, TWB only includes individuals aged 30–70 years, limiting the generalizability of the study results to other age groups. Second, because it is not a long-term follow-up data and only the most recent height measurement is used, this may not fully explain the decrease in height with age. Third, due to data limitations of TPMI, which has not yet completed imputed SNP data, we lack fully available imputed SNP data and we can only use SNPs confirmed by genotyping microarrays, which limits the genetic resolution of the study. In addition, although we validated the model using the Taiwan Precision Medicine Initiative (TPMI) dataset, both TWB and TPMI datasets are from the same population, which may limit generalization to other populations. Finally, although non-genetic factors such as year of birth and age at measurement improve prediction accuracy, they may introduce collinearity and overfitting risks, even if we checked for these issues.

In conclusion, the height prediction model which matches theoretical expectations has been effectively developed and validated within the Han Chinese population of both TWB and TPMI databases. We employed a 10-fold cross-validation procedure^33–36^ to ensure methodological rigor in developing and evaluating the final model. It has long been known that an increase in height is correlated with improved nutrition^56^ and a decrease in height is correlated with advanced age^57^. Despite potential variations in environmental and genetic factors across different databases, this study consistently emphasizes the predictability of height based on combining genetic factors, birth year, and age at measurement. It also underscores the high data quality of the two Taiwanese databases. Understanding the genetics of height carries significant importance, given its associations with various diseases. These newly established predictors for Han Chinese height represent another crucial step toward achieving this overarching research objective.

## Supplementary information

Supplementary

## Data Availability

The relevant data and summary statistics are publicly available at https://pheweb.ibms.sinica.edu.tw.
