# Supplementary material for: Polygenic height prediction for the Han Chinese in Taiwan
Source: NPJ Genom Med. 2025 Feb 5;10:7. doi: 10.1038/s41525-025-00468-6 (PMC11799370; doi:10.1038/s41525-025-00468-6)
Supplement: Supplementary file 1 — Supplementary [file 41525_2025_468_MOESM1_ESM.pdf]

Supplementary Table 1. Number of selected SNPs for height GWAS and LASSO in the TWB training set

	Female										
	**Training 1**	**Training 2**	**Training 3**	**Training 4**	**Training 5**	**Training 6**	**Training 7**	**Training 8**	**Training 9**	**Training 10**	**Intersecting**
**GWAS (p-value < 0.05)**	57,418	57,059	57,953	57,148	57,552	58,208	57,602	57,071	57,733	57,188	38,217
**LASSO**	29,454	31,806	32,124	29,779	29,024	30,435	27,682	30,525	32,261	29,823	**20,311**

	Male										
	**Training 1**	**Training 2**	**Training 3**	**Training 4**	**Training 5**	**Training 6**	**Training 7**	**Training 8**	**Training 9**	**Training 10**	**Intersecting**
**GWAS (p-value < 0.05)**	42,919	42,600	42,984	43,113	42,753	43,326	43,121	42,421	43,598	42,863	11,993
**LASSO**	9,981	8,687	9,345	9,579	9,243	9,108	9,359	10,081	10,124	9,551	**5,878**

GWAS: Genome-wide association studies

LASSO: Least absolute shrinkage and selection operator

Supplementary Table 2. The actual and predicted height distribution in TWB participants (N=78,719)

Model	N	Mean ± SD	Median (range)	Difference > 5% N (%) [a]	PCC	PCC's SD
**Male**						
**Actual**						
	24655	169.47 ± 6.29	169.5 (112.00 - 200.00)			
**Training set**						
**Birth year in AD**	221895	169.47 ± 2.52	169.26 (165.21 - 173.99)	3395 (13.77 %)	0.4001	0.0019
**Age at measurement**	221895	169.47 ± 2.50	169.23 (164.63 - 174.38)	3408 (13.82 %)	0.3968	0.0019
**Birth year + age at measurement**	221895	169.47 ± 2.52	169.25 (165.18 - 174.01)	3384 (13.73 %)	0.4001	0.0019
**Testing set**						
**Birth year in AD**	24655	169.47 ± 2.52	169.25 (165.18 - 174.02)	3403 (13.80 %)	0.3999	0.0058
**Age at measurement**	24655	169.47 ± 2.50	169.23 (164.65 - 174.41)	3409 (13.83 %)	0.3966	0.0058
**Birth year + age at measurement**	24655	169.47 ± 2.52	169.25 (165.16 - 174.05)	3401 (13.79 %)	0.3999	0.0058

Model	N	Mean ± SD	Median (range)	Difference > 5% N (%) [a]	PCC	PCC's SD
**Female**						
**Actual**						
	54064	157.43 ± 5.66	157.50 (118.50 - 181.50)			
**Training set**						
**Birth year in AD**	486576	157.43 ± 1.98	157.24 (153.82 - 161.23)	7305 (13.51 %)	0.3500	0.0013
**Age at measurement**	486576	157.43 ± 1.96	157.20 (153.26 - 161.53)	7307 (13.52 %)	0.3465	0.0013
**Birth year + age at measurement**	486576	157.43 ± 1.98	157.21 (153.76 - 161.27)	7266 (13.44 %)	0.3501	0.0013
**Testing set**						
**Birth year in AD**	54064	157.43 ± 1.98	157.24 (153.80 - 161.26)	7314 (13.53 %)	0.3498	0.0040
**Age at measurement**	54064	157.43 ± 1.96	157.20 (153.28 - 161.55)	7313 (13.53 %)	0.3464	0.0040
**Birth year + age at measurement**	54064	157.43 ± 1.98	157.21 (153.71 - 161.32)	7273 (13.45 %)	0.3499	0.0040

SD: standard deviation ; **AD:** Anno Domini ; **PCC:** Pearson correlation coefficient ; SD equation for PCC = sqrt [(1 - PCC²) / (N - 2)]

[a]: Number of samples with a value greater than 0.05 from take the absolute value after subtracting the actual height from the predicted height and then divided by the actual height.

Supplementary Table 3. TWB female and male principal component eigenvalue

Principal component	TWB Female PCA Eigenvalue	TWB Male PCA Eigenvalue
**1**	109.225	15.3310
**2**	45.3249	8.1037
**3**	26.7640	5.5346
**4**	18.2338	4.1983
**5**	13.4210	3.3730
**6**	10.3647	2.8101
**7**	8.2678	2.4003
**8**	6.7482	2.0880
**9**	5.6013	1.8416
**10**	4.7078	1.6420
**11**	3.9941	1.4768
**12**	3.4121	1.3378
**13**	2.9293	1.2191
**14**	2.5231	1.1164
**15**	2.1769	**1.0267**
**16**	1.8788	0.9476
**17**	1.6197	0.8774
**18**	1.3926	0.8145
**19**	1.1920	0.7579
**20**	**1.0138**	0.7066
**21**	0.8544	0.6600
**22**	0.7112	0.6174
**23**	0.5818	0.5783
**24**	0.4644	0.5423
**25**	0.3575	0.5090

Supplementary Table 4. Actual and predicted height distribution of different Chinese ethnic groups in Taiwan biobank (TWB)

	Female			Male		
	Number	Mean ± SD	P-value *	Number	Mean ± SD	P-value *
**Actual height**						
**Mother Chinese ethnic groups**			0.4959			0.5663
**Min-Nan**	35146	157.37 ± 5.66		15599	169.36 ± 6.32	
**Hakka**	7167	156.84 ± 5.57		3404	168.60 ± 6.24	
**Mainlander**	3469	157.93 ± 5.70		1562	170.45 ± 6.06	
**Father Chinese ethnic groups**			0.0767			0.6112
**Min-Nan**	32208	157.24 ± 5.66		14255	169.19 ± 6.30	
**Hakka**	6637	156.76 ± 5.54		3196	168.53 ± 6.22	
**Mainlander**	7033	158.31 ± 5.63		3178	170.76 ± 6.12	
**Training set 'PGS + birth year in AD + age at measurement' height prediction**						
**Mother Chinese ethnic groups**			0.6204			0.4642
**Min-Nan**	35146	157.39 ± 4.87		15599	169.33 ± 5.51	
**Hakka**	7167	156.66 ± 4.83		3404	168.58 ± 5.45	
**Mainlander**	3469	158.21 ± 5.07		1562	170.72 ± 5.48	
**Father Chinese ethnic groups**			0.5042			0.3671
**Min-Nan**	32208	157.25 ± 4.88		14255	169.15 ± 5.49	
**Hakka**	6637	156.63 ± 4.79		3196	168.49 ± 5.39	
**Mainlander**	7033	158.42 ± 4.90		3178	170.93 ± 5.43	
**Testing set 'PGS + birth year in AD + age at measurement' height prediction**						
**Mother Chinese ethnic groups**			0.5340			0.4113
**Min-Nan**	35146	157.40 ± 6.06		15599	169.31 ± 5.69	
**Hakka**	7167	156.59 ± 6.94		3404	168.57 ± 5.60	
**Mainlander**	3469	158.37 ± 31.29		1562	170.86 ± 5.82	
**Father Chinese ethnic groups**			0.4949			0.4083
**Min-Nan**	32208	157.25 ± 5.90		14255	169.14 ± 5.66	
**Hakka**	6637	156.55 ± 5.38		3196	168.47 ± 5.56	
**Mainlander**	7033	158.6 ± 22.98		3178	171.02 ± 5.71	

AD: Anno Domini, SD : standard deviation

*: P-value for ANOVA test

Supplementary Table 5. The relationship between bone density T-score or Z-score and birth year in AD or age at measurement in TWB participants

	Female						Male					
	T-score			Z-score			T-score			Z-score		
	Estimate	SD	P-value *	Estimate	SD	P-value*	Estimate	SD	P-value*	Estimate	SD	P-value*
**Birth year in AD**	0.00341	0.00382	0.3721	0.00402	0.00380	0.2907	0.00092	0.00490	0.8503	0.00088	0.00489	0.8569
**Age at measurement**	-0.06786	0.00380	<0.0001	-0.01227	0.00379	0.0012	-0.03206	0.00487	<0.0001	-0.00193	0.00486	0.6909

AD: Anno Domini, **SD**: standard deviation

*: P-value for linear regression

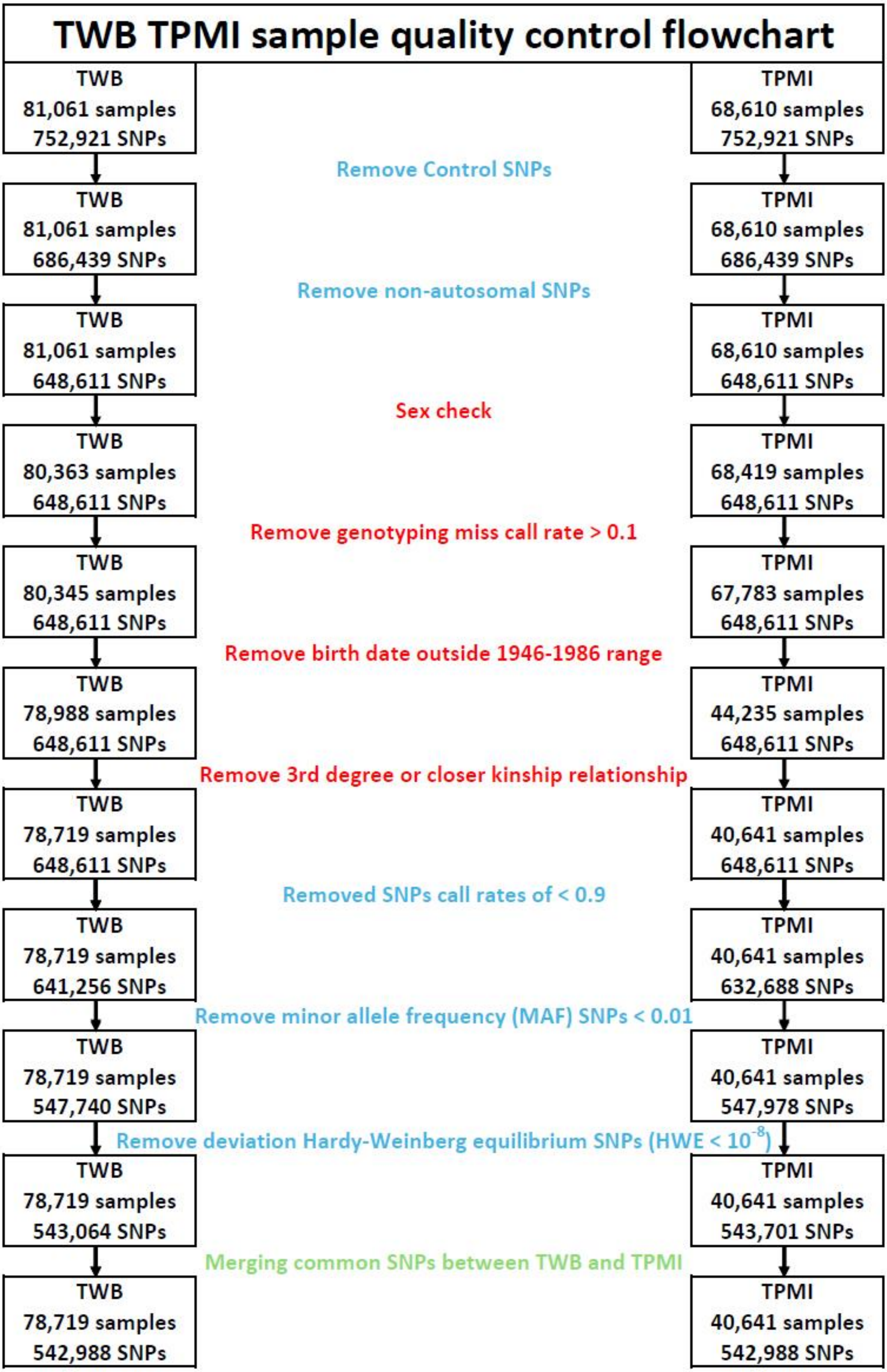

Supplementary Figure 1. TWB TPMI sample quality control flow chard

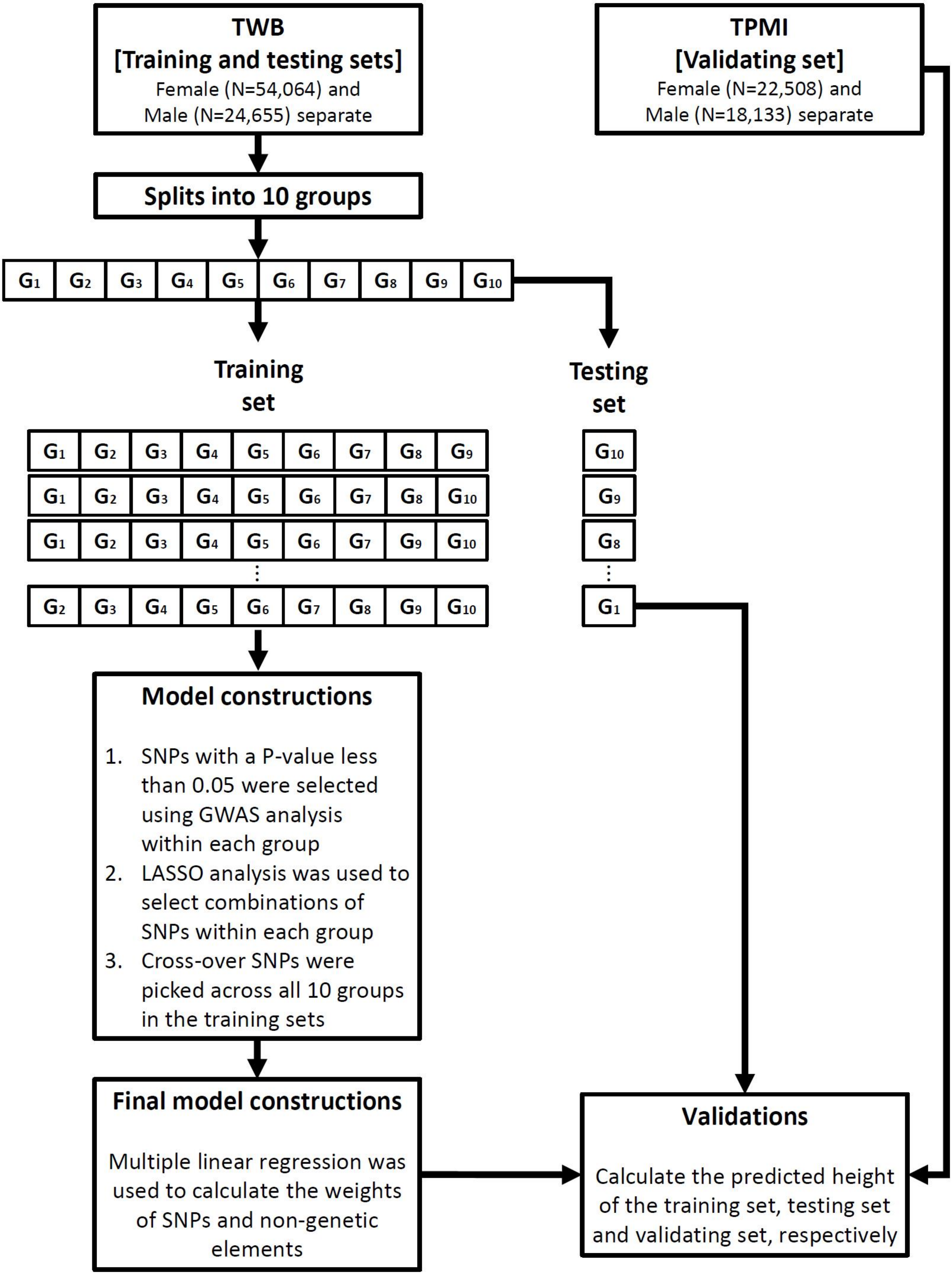

Supplementary Figure 2. Height predictor construction flow chart

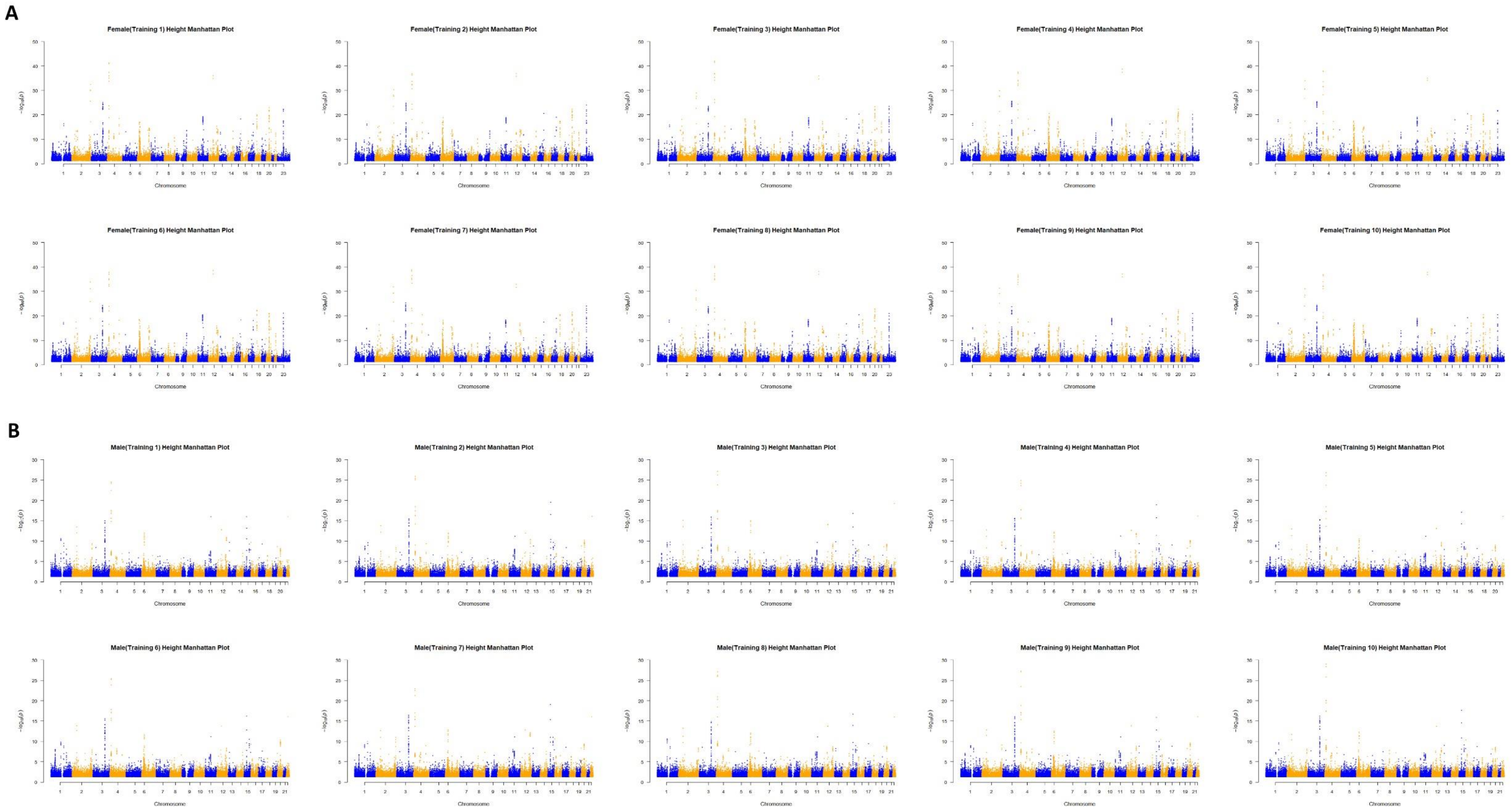

Supplementary Figure 3. Genome-wide association analysis (GWAS) Manhattan plot results for males and females in the TWB training set
(A) Female, (B) Male

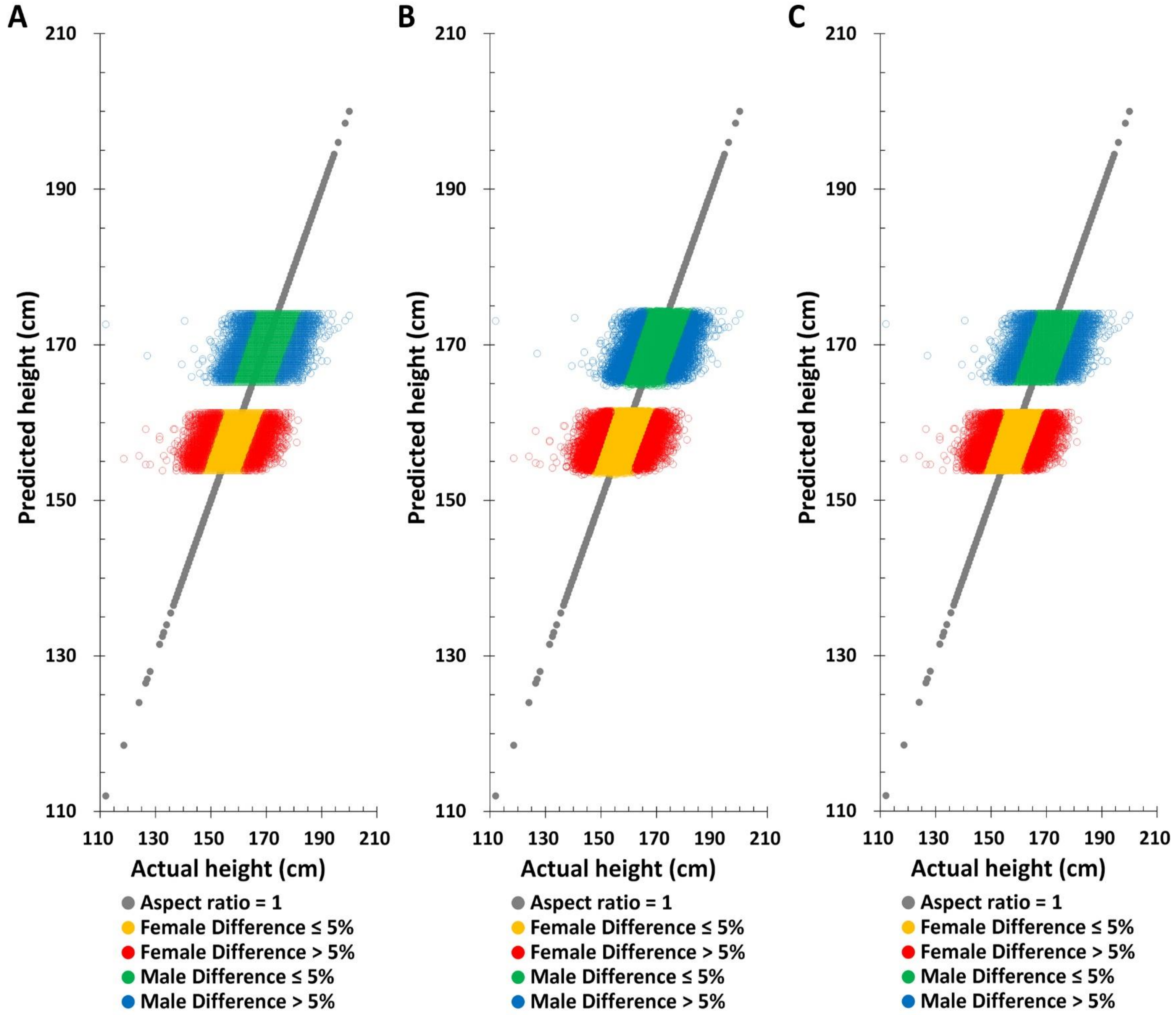

Supplementary Figure 4. The distribution of actual height and predicted height based on different factors combination in the Taiwan Biobank training set

(A) Birth year in AD only, (B) Age at measurement only, (C) Birth year in AD + Age at measurement.

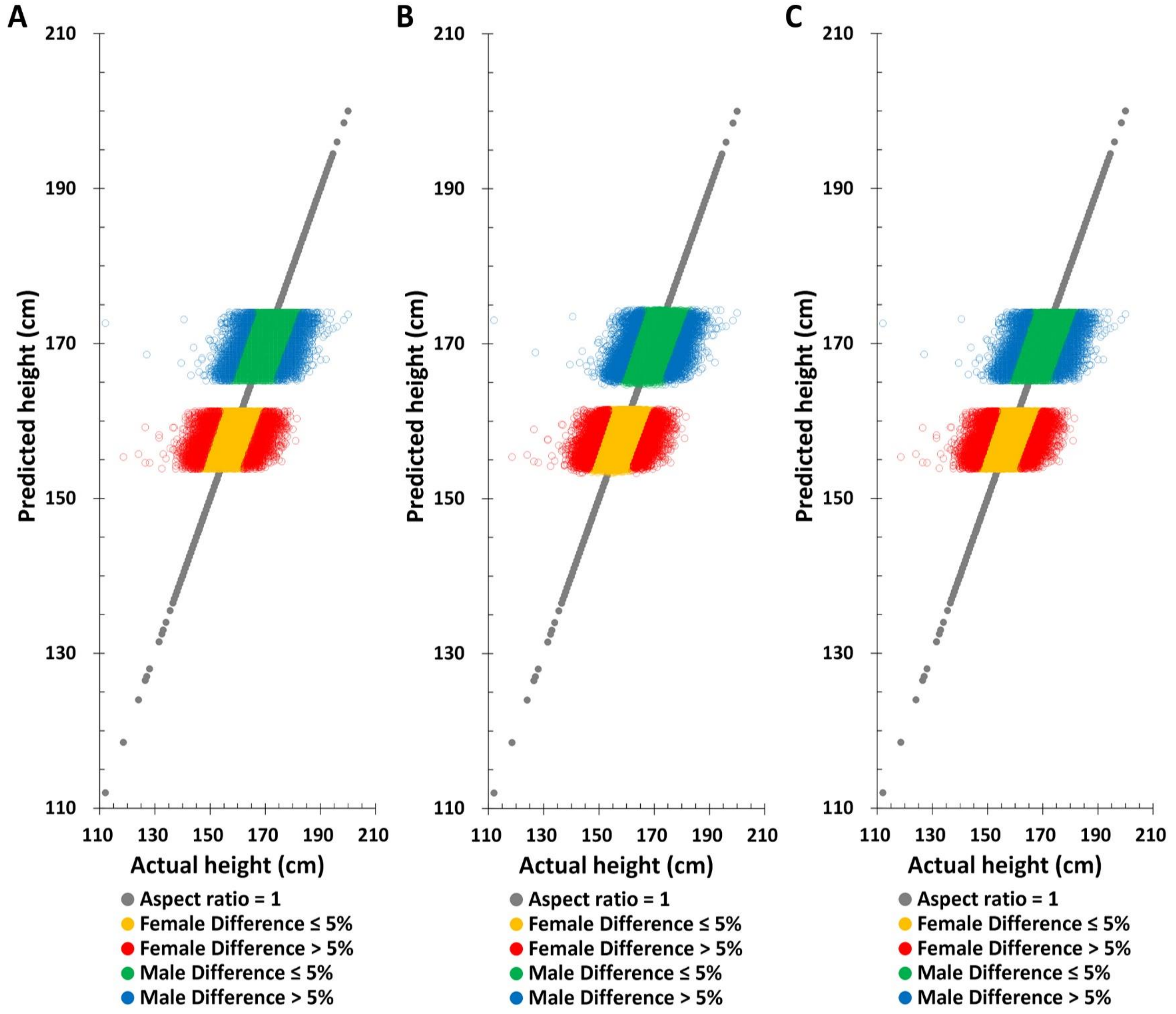

Supplementary Figure 5. The distribution of actual height and predicted height based on different factors combination in the Taiwan Biobank testing set

(A) Birth year in AD only, (B) Age at measurement only, (C) Birth year in AD + Age at measurement.

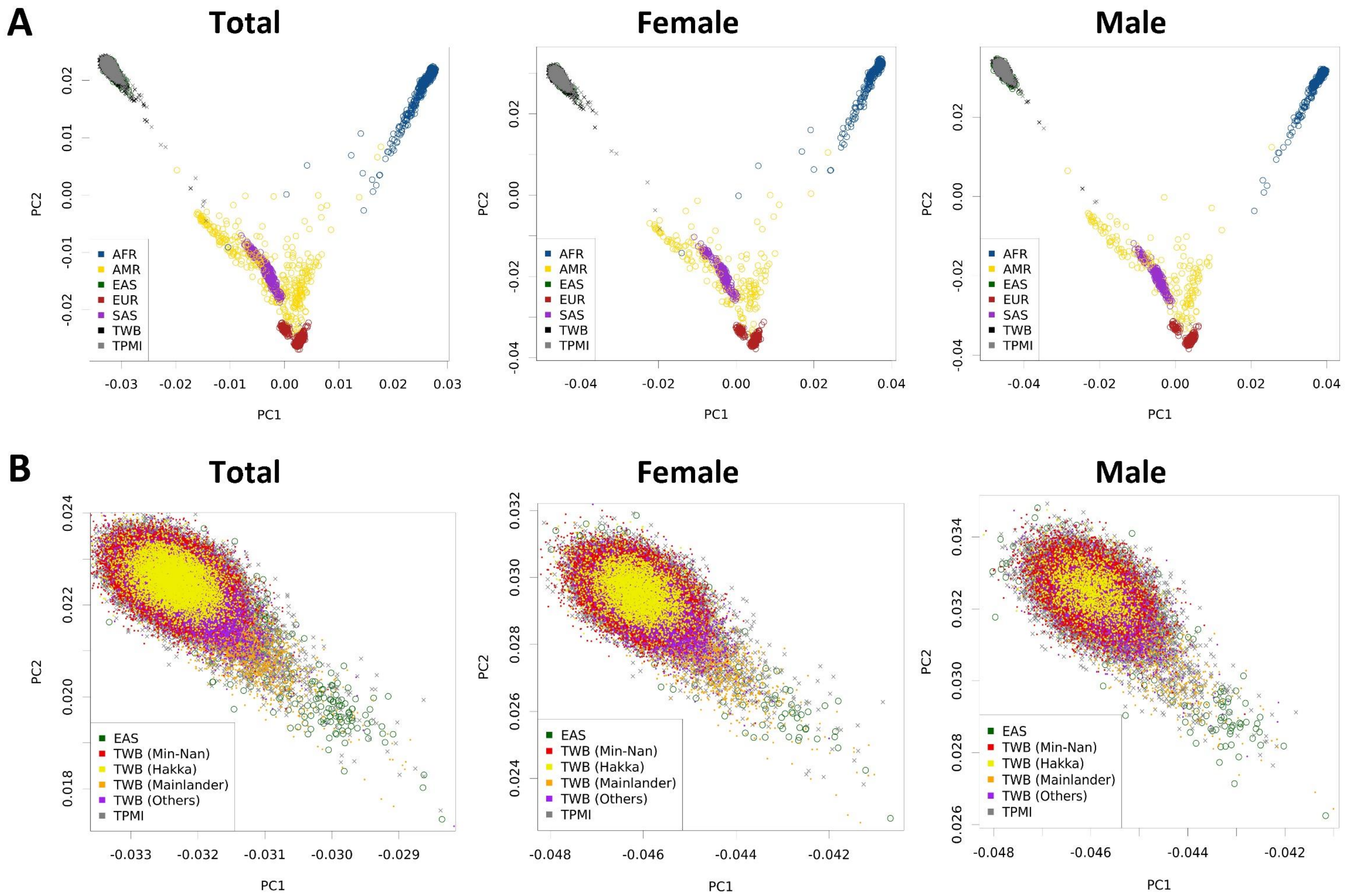

Supplementary Figure 6. PCA clustering results for TWB and TPMI samples and the 1000 Genomes Project
(A) TWB and TPMI samples and the 1000 Genomes, (B) Classified by ancestry in TWB and TPMI samples and the 1000 Genomes East Asian ancestry population. **AFR:** African ; **AMR:** Ad Mixed American ; **EAS:** East Asian ; **EUR:** European ; **SAS:** South Asian

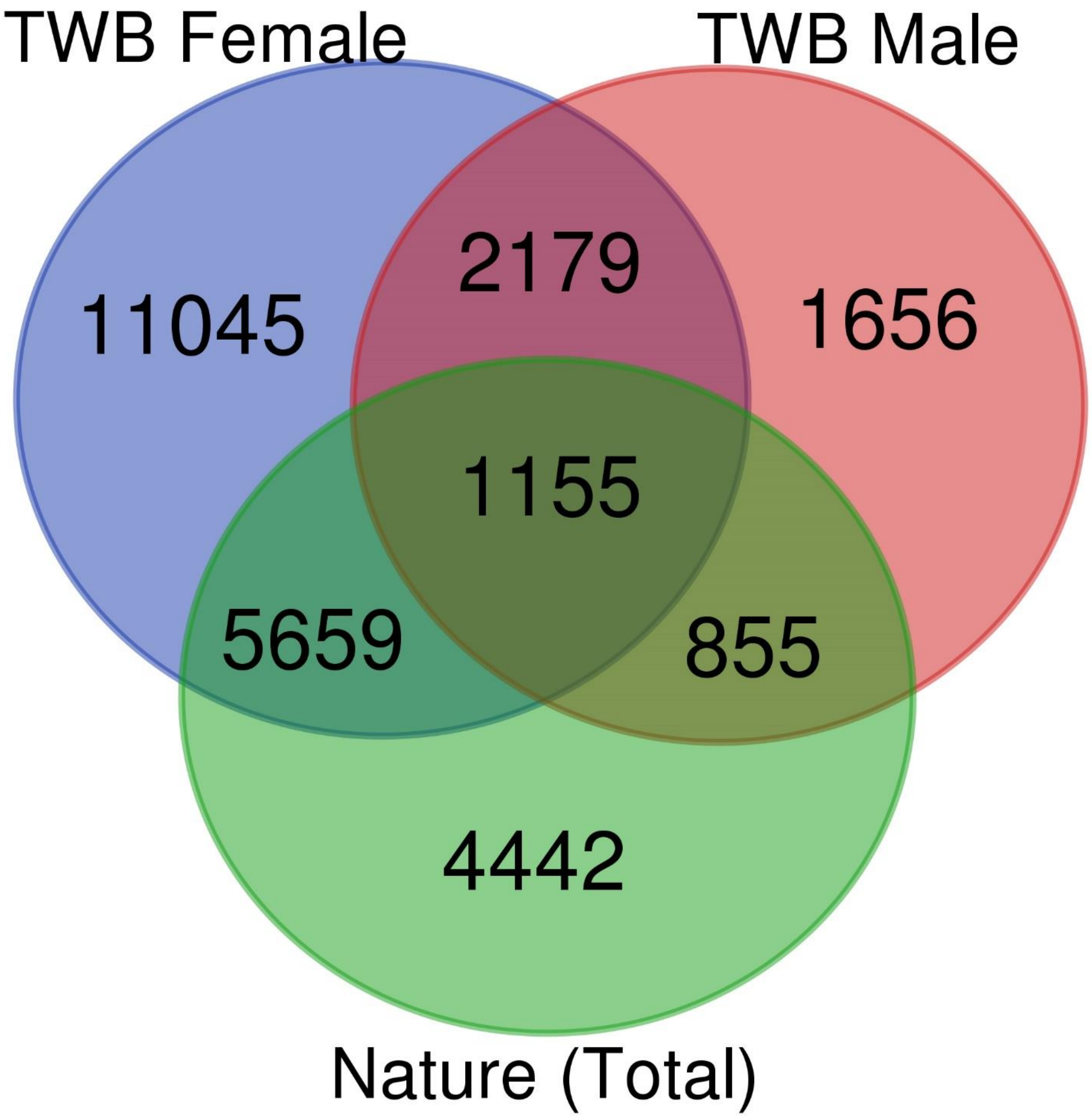

Supplementary Figure 7. Venn diagram of SNPs used for height prediction in females and males in this study compared to SNPs used for height prediction in Yengo et al. (2022)

This diagram shows the overlap between the SNPs selected for height prediction in females and males in our study and the SNPs identified for height prediction in the Yengo et al. (2022) study. The diagram illustrates the proportion of shared SNPs and those unique to each dataset.

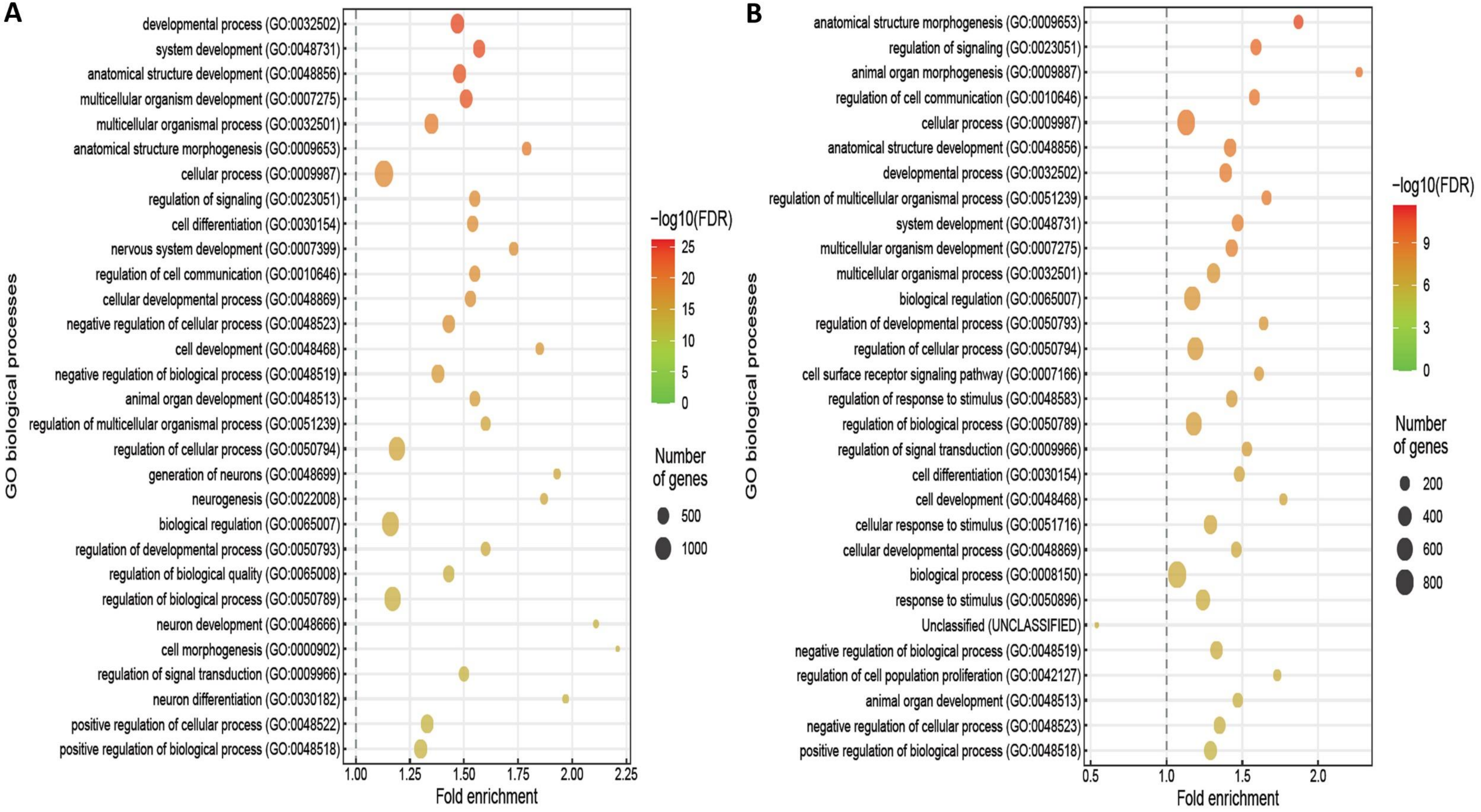

Supplementary Figure 8. Gene Ontology functional analysis

GO functional analysis clustered the height related genes in (A) female and (B) male, respectively. The most representative and significant biological processes were represented and were sorted by false discovery rate (FDR). The dot size indicated the number of genes associated with the process and the dot color indicated the significance of the enrichment (-log10(FDR-corrected *P*-values)). The vertical grey dashed line represented a fold enrichment of 1.
